# Supplementary material for: Enzalutamide induces cytotoxicity in desmoplastic small round cell tumor independent of the androgen receptor
Source: Commun Biol. 2024 Apr 4;7:411. doi: 10.1038/s42003-024-06003-0 (PMC10995187; doi:10.1038/s42003-024-06003-0)
Supplement: Supplementary file 5 — Reporting Summary [file 42003_2024_6003_MOESM5_ESM.pdf]

nature portfolio

Corresponding author(s): Sean B. Lee

Last updated by author(s): Nov 28, 2023

# Reporting Summary

Nature Portfolio wishes to improve the reproducibility of the work that we publish. This form provides structure for consistency and transparency in reporting. For further information on Nature Portfolio policies, see our Editorial Policies and the Editorial Policy Checklist.

## Statistics

For all statistical analyses, confirm that the following items are present in the figure legend, table legend, main text, or Methods section.

| n/a | Confirmed | |
|---|---|---|
| ☐ | ☒ | The exact sample size (*n*) for each experimental group/condition, given as a discrete number and unit of measurement |
| ☐ | ☒ | A statement on whether measurements were taken from distinct samples or whether the same sample was measured repeatedly |
| ☐ | ☒ | The statistical test(s) used AND whether they are one- or two-sided<br>*Only common tests should be described solely by name; describe more complex techniques in the Methods section.* |
| ☒ | ☐ | A description of all covariates tested |
| ☒ | ☐ | A description of any assumptions or corrections, such as tests of normality and adjustment for multiple comparisons |
| ☐ | ☒ | A full description of the statistical parameters including central tendency (e.g. means) or other basic estimates (e.g. regression coefficient) AND variation (e.g. standard deviation) or associated estimates of uncertainty (e.g. confidence intervals) |
| ☐ | ☒ | For null hypothesis testing, the test statistic (e.g. $F$, $t$, $r$) with confidence intervals, effect sizes, degrees of freedom and $P$ value noted<br>*Give P values as exact values whenever suitable.* |
| ☒ | ☐ | For Bayesian analysis, information on the choice of priors and Markov chain Monte Carlo settings |
| ☒ | ☐ | For hierarchical and complex designs, identification of the appropriate level for tests and full reporting of outcomes |
| ☒ | ☐ | Estimates of effect sizes (e.g. Cohen's *d*, Pearson's *r*), indicating how they were calculated |

*Our web collection on statistics for biologists contains articles on many of the points above.*

## Software and code

Policy information about availability of computer code

| | |
|---|---|
| Data collection | N/A |
| Data analysis | N/A |

For manuscripts utilizing custom algorithms or software that are central to the research but not yet described in published literature, software must be made available to editors and reviewers. We strongly encourage code deposition in a community repository (e.g. GitHub). See the Nature Portfolio guidelines for submitting code & software for further information.

## Data

Policy information about availability of data

All manuscripts must include a data availability statement. This statement should provide the following information, where applicable:

- Accession codes, unique identifiers, or web links for publicly available datasets
- A description of any restrictions on data availability
- For clinical datasets or third party data, please ensure that the statement adheres to our policy

Data was uploaded to GEO and can be accessed at GSE245914

# Research involving human participants, their data, or biological material

Policy information about studies with human participants or human data. See also policy information about sex, gender (identity/presentation), and sexual orientation and race, ethnicity and racism.

| | |
|---|---|
| Reporting on sex and gender | Gender is reported in Figure 6E. |
| Reporting on race, ethnicity, or other socially relevant groupings | This data was not available for reporting. |
| Population characteristics | Age is reported in Figure 6E. |
| Recruitment | Patient recruitment was based on having a diagnosis of DSRCT and being treated at MD Anderson Cancer Center. |
| Ethics oversight | MDACC's Institutional Review Board |

Note that full information on the approval of the study protocol must also be provided in the manuscript.

# Field-specific reporting

Please select the one below that is the best fit for your research. If you are not sure, read the appropriate sections before making your selection.

☒ Life sciences ☐ Behavioural & social sciences ☐ Ecological, evolutionary & environmental sciences

For a reference copy of the document with all sections, see nature.com/documents/nr-reporting-summary-flat.pdf

# Life sciences study design

All studies must disclose on these points even when the disclosure is negative.

| | |
|---|---|
| Sample size | Sample size of at least 2 was used for western blots and at least 3 for other experiments. |
| Data exclusions | No data were excluded for the analyses. |
| Replication | Add results were independently replicated multiple times. Four independent shRNAs were used for AR knockdown to ensure scientific rigor. |
| Randomization | For tumor seeding experiments, mice were randomized to receive cells from adherent or sphere culture conditions. |
| Blinding | No blinding was used in these experiments. |

# Reporting for specific materials, systems and methods

We require information from authors about some types of materials, experimental systems and methods used in many studies. Here, indicate whether each material, system or method listed is relevant to your study. If you are not sure if a list item applies to your research, read the appropriate section before selecting a response.

## Materials & experimental systems

| n/a | Involved in the study | |
|---|---|---|
| ☐ | ☒ | Antibodies |
| ☐ | ☒ | Eukaryotic cell lines |
| ☒ | ☐ | Palaeontology and archaeology |
| ☐ | ☒ | Animals and other organisms |
| ☒ | ☐ | Clinical data |
| ☒ | ☐ | Dual use research of concern |
| ☒ | ☐ | Plants |

## Methods

| n/a | Involved in the study | |
|---|---|---|
| ☒ | ☐ | ChIP-seq |
| ☒ | ☐ | Flow cytometry |
| ☒ | ☐ | MRI-based neuroimaging |

## Antibodies

| | |
|---|---|
| Antibodies used | Antibody Company Catalog # WB Dilution<br>β-Actin (8H10D10) Cell Signaling 3700 1:1000<br>AR Cell Signaling D6F11 1:1000<br>LCK Cell Signaling L22B1 1:500 |

EWSR1 (N-term) Lab Created N/A 1:1000
MERTK Cell Signaling 348E6 1:1000
FLAG Sigma F3165 1:1000

Validation

All antibodies have been validated by the manufacturer and in our own hands by western blot analysis in cells of known protein expression.

## Eukaryotic cell lines

Policy information about cell lines and Sex and Gender in Research

Cell line source(s)

JN-DSRCT-1, BER-DSRCT, BOD-DSRCT, LP9

Authentication

DSRCT ell lines have been described previously and validated for the defining EWSR1::WT1 fusion (via PCR and sequencing).

Mycoplasma contamination

Cell lines were regularly tested for mycoplasma contamination.

Commonly misidentified lines
(See ICLAC register)

N/A

## Animals and other research organisms

Policy information about studies involving animals; ARRIVE guidelines recommended for reporting animal research, and Sex and Gender in Research

Laboratory animals

Male or Female NOD-SCID-IL2Rγ-null (NSG) mice (6 weeks) were purchased (Jackson Laboratory, Bar Harbor, ME) and used for all xenograft studies

Wild animals

N/A

Reporting on sex

Sex as a variable of interest in our studies. We performed identical tumor seeding in male and female mice to test the role of androgen in xenograft growth.

Field-collected samples

N/A

Ethics oversight

Animal procedures were approved by the Tulane Institutional Animal Care and Use Committee (Protocol: 1500) OR University of Texas MD Anderson Cancer Center (MDACC; Houston, TX) Institutional Animal Care and Use Committee (eACUF Protocols #00000712-RN02)

Note that full information on the approval of the study protocol must also be provided in the manuscript.

## Plants

Seed stocks

*Report on the source of all seed stocks or other plant material used. If applicable, state the seed stock centre and catalogue number. If plant specimens were collected from the field, describe the collection location, date and sampling procedures.*

Novel plant genotypes

*Describe the methods by which all novel plant genotypes were produced. This includes those generated by transgenic approaches, gene editing, chemical/radiation-based mutagenesis and hybridization. For transgenic lines, describe the transformation method, the number of independent lines analyzed and the generation upon which experiments were performed. For gene-edited lines, describe the editor used, the endogenous sequence targeted for editing, the targeting guide RNA sequence (if applicable) and how the editor was applied.*

Authentication

*Describe any authentication procedures for each seed stock used or novel genotype generated. Describe any experiments used to assess the effect of a mutation and, where applicable, how potential secondary effects (e.g. second site T-DNA insertions, mosiacism, off-target gene editing) were examined.*
